# Cheerfulness

**Published:** 1854-09

**Authors:** 


### CHEERFULNESS.

Reader, if you would be loved by those around you, be cheerful,—it will be like sunshine upon the clouds. Cheerfulness qualifies us the better for society, promotes health, enlivens beauty, lengthens life, and increases usefulness. A cheerful face may hide a bleeding heart; but grief ought sometimes to be forgotten in the desire to hide our own sorrows and bring sunshine instead of shade to those we meet;—thus we shed a pure ray of joy on our own path, and lighten life's journey to others. Be buoyant, then,—look on the bright side of the picture—cheer up yourself and those around you too. What a treasure is a house made happy by each member of the family contributing a pleasant smile and a cheerful look,—bringing peace and quietude and gladness where else contentions reign. The writer has often felt that the happiest hours of her life were those spent at the evening fireside, surrounded by her little family, engaged with book or work, teaching them cheerfulness, contentment, and piety—thus preparing them for usefulness here, for a dying hour, and the Judgment Day!

